# Organ dysfunction and mortality in septic patients admitted to an ICU

**DOI:** 10.1186/cc12950

**Published:** 2013-11-05

**Authors:** Thais Almeida Rodrigues, Louise Cristhine de Carvalho Santos, Jaqueline Lima de Souza, Fernanda Vilas Bôas Araújo, Thiago Alves Silva, Pedro Henrique Gomes Rocha, Lucila de Jesus Almeida, Jacqueline Rodrigues de Carvalho, Mariana Pinheiro Barbosa de Araújo, Lucas Garcia de Souza Godoy, Kátia Crys Moura Ogliari, Pedro Nery Ferreira Júnior, Adriell Ramalho Santana, Fábio Ferreira Amorim, Clayton Barbieri de Carvalho

**Affiliations:** 1 Escola Superior de Ciências da Saúde, Brasília, Brazil; 2 Liga Acadêmica de Medicina Intensiva de Brasília, Brazil; 3 Hospital Regional de Samambaia, Brasília, Brazil

## Background

Sequential Organ Failure Assessment (SOFA) is a largely used score in the evaluation of organ dysfunction/failure in septic patients [1]. Repeated scoring can also assess patient condition and disease development [2]. The present study aims to describe the association between SOFA score and the organ dysfunction components of this score with mortality in septic patients admitted to an ICU.

## Materials and methods

Prospective study conducted on patients admitted to the ICU of Hospital Regional de Samambaia, Brasília, DF, Brazil, during 7 months. The SOFA was evaluated at the time of admission to the ICU. Patients were divided into two groups: survivors group (SG) and nonsurvivors group (NSG). Accuracy of SOFA and the organ dysfunction components of SOFA score to predict ICU mortality were measured with the area under the receiver operating characteristic (ROC) curve.

## Results

**Figure 1:**
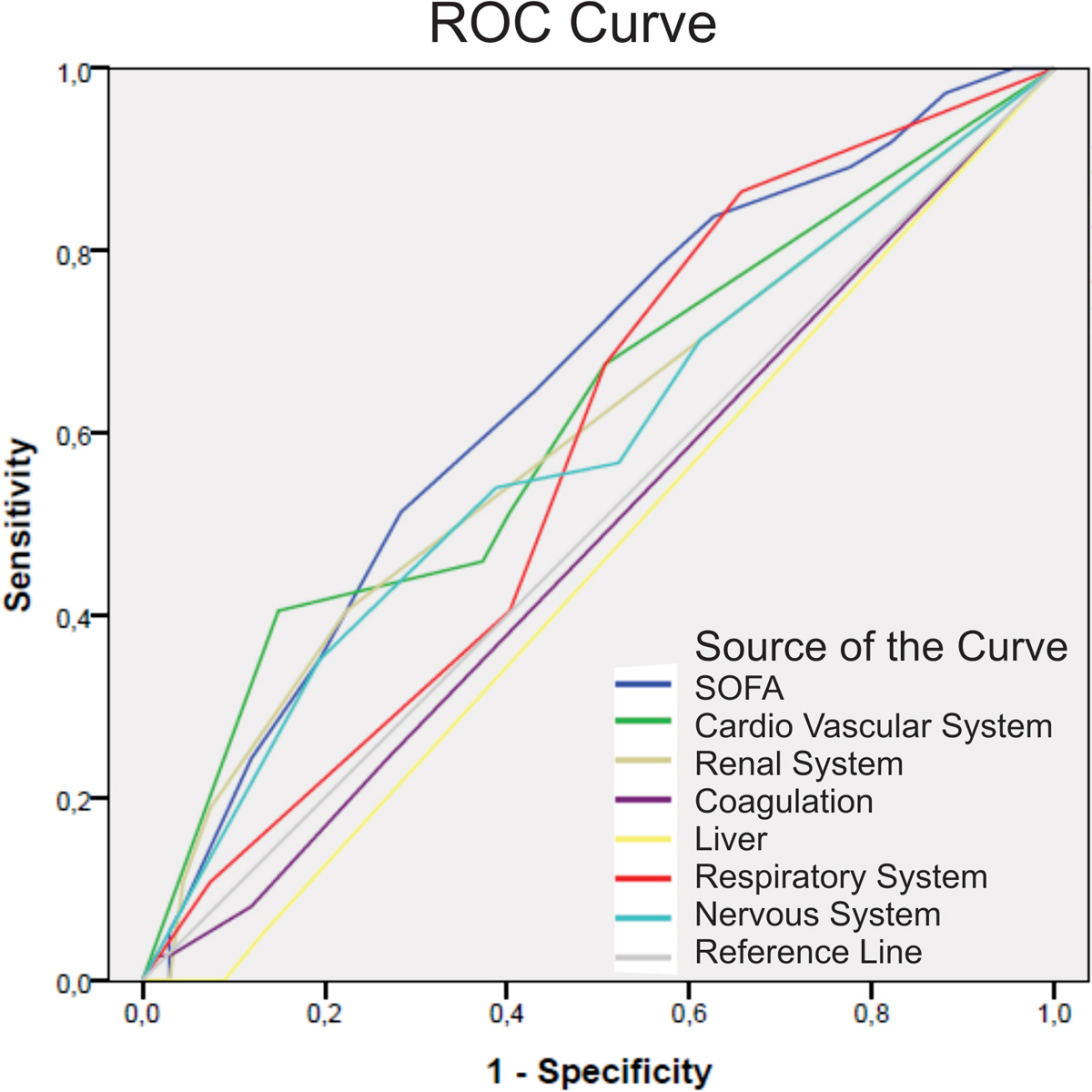
**ROC curve for SOFA**.

One hundred and seven patients were enrolled. Mean age was 53 ± 20, APACHE II 14 ± 6, SAPS 3 52.9 ± 14.9 and SOFA 6.2 ± 3.3. ICU mortality was 34.6% (*n *= 37). The SOFA score was higher in nonsurvivors (7.4 ± 3.0 vs. 5.8 ± 3.4, *P *= 0.01), cardiovascular (2.0 ± 1.8 vs. 1.4 ± 1.6, *P *= 0.01) and kidney dysfunctions (0.7 ± 1.0 vs. 0.4 ± 0.9, *P *= 0.04) being higher in this group. There were no differences between the groups regarding coagulation (0.4 ± 0.8 vs. 0.4 ± 0.8, *P *= 0.59), liver (0.0 ± 0.3 vs. 0.0 ± 0.7, *P *= 0.65), respiratory (2.0 ± 1.2 vs. 1.6 ± 1.4, *P *= 0.87), and neurologic (2.2 ± 1.7 vs. 1.7 ± 1.6, *P *= 0.96) organ dysfunctions. The area under the ROC curve (Figure 1) for SOFA was 0.650 (95% CI 0.541 to 0.759). The components of the cardiovascular system, renal system, coagulation, liver, respiratory, and nervous systems had areas under the ROC curve of 0.612 (95% CI 0.501 to 0.732), 0.565 (95% CI 0.478 to 0.712), 0.484 (95% CI 0.369 to 0.600), 0.457 (95% CI 0.344 to 0.571), 0.580 (95% CI 0.469 to 0.691), and 0.582 (95% CI 0.465 to 0.699), respectively.

## Conclusions

The SOFA score was moderately associated with ICU mortality. The scores for cardiovascular and renal impairment were individually associated with mortality.
